# Identification of Patients At-Risk of QT Interval Prolongation during Medication Reviews: A Missed Opportunity?

**DOI:** 10.3390/jcm7120533

**Published:** 2018-12-10

**Authors:** Vera H. Buss, Kayla Lee, Mark Naunton, Gregory M. Peterson, Sam Kosari

**Affiliations:** 1 Faculty of Health, University of Canberra, Bruce, ACT 2617, Australia; Vera.Buss@canberra.edu.au (V.H.B.); kaylalee_@live.com.au (K.L.); Mark.Naunton@canberra.edu.au (M.N.); G.Peterson@utas.edu.au (G.M.P.); 2 School of Medicine, University of Tasmania, Sandy Bay, TAS 7005, Australia

**Keywords:** QT interval prolongation, torsade de pointes, medication review, pharmacist intervention

## Abstract

The prolongation of the QT interval is a relatively rare but serious adverse drug reaction. It can lead to torsade de pointes, which is potentially life-threatening. The study’s objectives were: determine the use of QT interval-prolonging drugs in an elderly community-dwelling population at risk of medication misadventure and identify recommendations regarding the risk of QT interval prolongation made by pharmacists when performing medication reviews. In a retrospective evaluation, 500 medication review reports from Australian pharmacists were analysed. In patients taking at least one QT interval-prolonging drug, the individual risk of drug-induced QT interval prolongation was assessed. Recommendations of pharmacists to avoid the occurrence of this drug-related problem were examined. There was a high prevalence of use of potentially QT interval-prolonging drugs (71% patients), with 11% of patients taking at least one drug with a known risk. Pharmacists provided specific recommendations in only eight out of 35 patients (23%) with a high-risk score and taking drugs with known risk of QT interval prolongation. Pharmacists’ recommendations, when present, were focused on drugs with known risk of QT interval prolongation, rather than patients’ additional risk factors. There is a need to improve knowledge and awareness of this topic among pharmacists performing medication reviews.

## 1. Introduction

A rare but serious adverse drug reaction is the prolongation of the QT interval, meaning that the section between the Q and T waves in the electrocardiogram is extended [1]. This effect is at least partially caused by a blockade of the outward rapid potassium current, leading to extended repolarisation. This may trigger an early after-depolarisation, which in combination with a heterogenous intracardiac repolarisation, is associated with the onset of torsade de pointes (TdP). TdP is a polymorphic ventricular tachycardia which can be seen in the electrocardiogram as twisting QRS complexes, which are responsible for the naming. It can result in sudden cardiac death [1].

Pharmaceutical companies are legally obliged to test new drugs for a potential QT interval-prolonging effect before gaining regulatory approval [2]. Certain drug classes are well known to include agents that can induce QT interval prolongation; these include antidepressants, non-sedating antihistamines, antimicrobials, antipsychotics, and cardiac drugs [3]. There is also an association between the plasma concentrations of these drugs and the risk of QT interval prolongation [4]. Therefore, clinicians must ensure appropriate dose adjustment in patients with chronic kidney or liver disease to avoid accumulation of QT interval-prolonging drugs [4]. Furthermore, problems can occur due to drug-drug interactions [4]. In particular, the combination of QT interval-prolonging drugs that are metabolised via cytochrome P450 (CYP450) with CYP450-inhibitors can lead to substantial plasma level increases [4]. 

In most cases, a combination of different risk factors is responsible for QT interval prolongation [5]. Besides drugs, some patient-specific risk factors include female gender, increasing age, pre-existing heart disease, and electrolyte imbalances (particularly hypokalaemia) [5].

The risk of drug-induced QT interval prolongation appears to be frequently overlooked in clinical practice [6]. Pharmacists are in the ideal position to identify and prevent this risk while conducting medication reviews [7]. In Australia, accredited pharmacists can perform Government-funded medication management reviews (Home Medicines Reviews; HMRs), in collaboration with general practitioners (GPs), for individuals living in the community who are at risk of medication misadventure [8]. First, the GP selects an eligible patient and refers them to an accredited pharmacist [8]. The pharmacist will visit the patient in their residence to perform the review [8]. Then, the pharmacist writes a report to the GP with recommendations [8]. Finally, the GP meets with the patient to establish an individualised medication plan and implement changes if necessary [8].

This study explored the role of pharmacists in identifying and reducing the risk of drug-induced QT interval prolongation when conducting medication reviews. The aim was to determine the prevalence of use of QT interval-prolonging drugs in an elderly community-dwelling population at risk of medication misadventure and to identify recommendations made by accredited pharmacists regarding QT interval prolongation in medication review reports. 

## 2. Experimental Section

The researchers retrospectively analysed 500 de-identified HMR reports. The HMRs were performed by nine accredited pharmacists between March 2011 and March 2015. The 500 reports were a random sample of all the reports of a medication review provider during that period. Patients were eligible for HMRs according to standard criteria, including taking at least five medications, suspected non-adherence, recent hospitalisation, or requiring education on the use of medicines [9]. 

The data extraction was performed in duplicate by two researchers (K.L. and V.H.B). Data regarding the patient’s demographics, the risk factors for QT interval prolongation, QT interval-prolonging drugs taken, and any recommendations made by the pharmacist regarding QT interval prolongation were extracted if the patient took at least one QT interval-prolonging drug. The CredibleMeds^®^ website (status as of 2015) was used to classify medications into three categories regarding their potential for inducing QT interval prolongation. ‘Known risk’ category includes drugs that prolong the QT interval and there is strong evidence of increasing the risk of TdP [10]. ‘Possible risk’ category includes drugs that can cause QT interval prolongation but lack evidence for a risk of TdP [10]. ‘Conditional risk’ category includes drugs that under certain circumstances (such as drug interaction, excessive dose, electrolyte disturbances) can be associated with TdP [10]. The fourth category of the CredibleMeds^®^ website, drugs that should be avoided in individuals with congenital long QT syndrome [10], was not assessed in this study. Some examples of drugs listed on the website are given in Table 1.

For the overall risk assessment for QT interval prolongation, the following factors were evaluated: increased age (>65 years), female gender, smoking status, obesity (body mass index ≥30 kg/m^2^), cardiomyopathy, hypertension, arrhythmia, prolonged QT interval, thyroid disturbance, liver failure, neurological disorders, diabetes, electrolyte imbalances (potassium ≤3.5 mmol/L, calcium <2.15 mmol/L), inflammation (C-reactive protein >5 mg/L), significant renal impairment (eGFR ≤30 mL/min/1.73 m^2^), and drugs with risk of QT interval prolongation [11,12]. In patients taking at least one QT interval-prolonging drug, individual risk was assessed using these factors within the RISQ-PATH score (Table 2) [12]. This is a validated tool to predict the risk of QT interval prolongation and ranges from 0 (low risk) to 40.5 (high risk) for patients’ specific risk factors plus the sum of the score associated with QT interval-prolonging drugs [12]. Patients with a score of 10 or above are considered as being at high risk of developing drug-induced QT interval prolongation [12]. The ACT Health Human Research Ethics Committee had granted ethics approval for the project (ETHLR.15.116). 

## 3. Results

**Figure 1:**
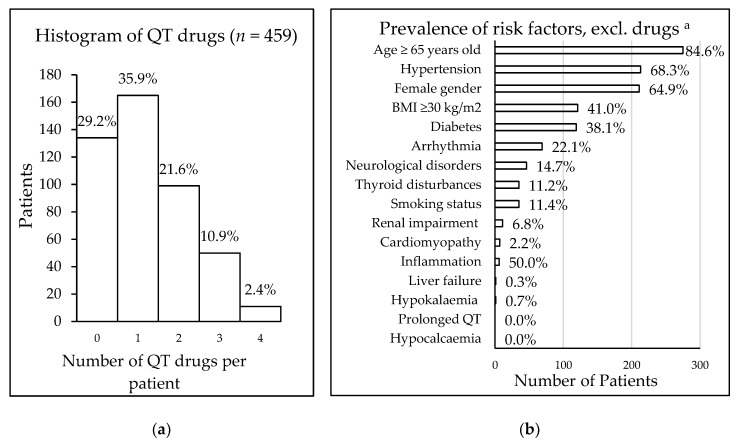
(**a**) Frequency of patients taking QT interval-prolonging drugs; (**b**) prevalence of additional risk factors for 325 patients taking QT interval-prolonging drugs, ^a^ data availability: age and gender *n* = 325, medical history *n* = 312, smoking *n* = 306, BMI *n* = 295, renal status *n* = 161, potassium level *n* = 151, calcium level *n* = 13, inflammation (CRP level) *n* = 12. Abbreviation: BMI = body mass index.

A total of 500 HMR reports were analysed. After duplicate reports (*n* = 41) were excluded, 459 HMR reports remained. In these 459 patients, 11.3% (52/459) were taking at least one drug with known risk, 13.5% (62/459) were taking at least one drug with a possible risk, and 63.4% (291/459) were taking at least one drug with a conditional risk. Overall, 325 patients (70.8%) were taking at least one drug associated with the risk of QT interval prolongation (Table 3). Their mean age was 76 (± 12) years. The most commonly prescribed QT interval-prolonging drug overall was hydrochlorothiazide, which is associated with conditional risk (90 of 459 reviewed patients; 19.6%), while citalopram and escitalopram were the most frequently taken known risk drugs, each with a prevalence of 2.8% (13/459) (Table 3). Eleven patients took a maximum of four QT interval-prolonging drugs (Figure 1). In total, 557 drugs were being taken that carried some risk of QT interval prolongation, including drugs with known risk (9.9%), possible risk (12.4%), and conditional risk (77.7%). 

The risk of drug-induced QT interval prolongation, using the RISQ-PATH score, was calculated for those 325 patients taking QT interval-prolonging drugs. The mean score was 9.4 (± 3.4); 49.5% of the patients had a score between 6 and 10 (161/325), and 42.2% of patients (137/325) had a score of 10 or higher, which was defined as high risk. Of all patients taking QT interval-prolonging drugs, 98.9% (321/325) had at least one patient-specific risk factor for QT interval prolongation, with older age being the most common of these (*n* = 275). The prevalence of various patient-specific risk factors is displayed in Figure 1. 

Out of 35 patients with a high-risk RISQ-PATH score of ≥10 and taking drugs having a known risk of QT interval prolongation, pharmacists provided specific recommendations regarding QT interval prolongation in only eight cases (23%). In a further eight cases (23%) they provided unspecific advice (e.g., risk of interaction, not suitable for patient, or recommended dose reduction), in six cases (17%) they mentioned a different potential adverse effect of the drug, and in the remaining 13 cases (37%) the pharmacists did not make any recommendation. It should be noted that donepezil was added to the CredibleMeds^®^ website in March 2015. That means that these results included one medication review report with unspecific advice regarding donepezil and two reports without any recommendations regarding donepezil, that were written before the drug was listed as “known risk”.

Overall, in 15 of 325 HMR reports (4.6%) for patients taking potential QT interval-prolonging drugs, the pharmacists specifically mentioned patients being at risk of QT interval prolongation. The comments were regarding excessive doses of specific drugs (citalopram *n* = 2, escitalopram *n* = 2, domperidone *n* = 2), prescription of specific drugs (tricyclic antidepressants *n* = 5, sotalol *n* = 1), and pharmacotherapy with several QT interval-prolonging drugs concomitantly (*n* = 9); some recommendations comprised several of these aspects. The suggested interventions were monitoring patients closely (*n* = 5), monitoring electrolytes (*n* = 2), change of drug (*n* = 4), withdrawal (*n* = 4), dose reduction (*n* = 4), and specialist referral (*n* = 3); for some patients several interventions were suggested. The median risk score for QT interval prolongation in these 15 patients was 11.25 (ranging from 1.75 to 16.25).

## 4. Discussion

Overall, 71% of medication review recipients were taking at least one drug associated with the risk of QT interval prolongation, including 11% taking at least one drug with known risk; yet, pharmacists’ recommendations relating to this risk were uncommon. Examples of implicated drugs included citalopram, escitalopram, domperidone, and tricyclic antidepressants. The former three have a high risk of QT interval prolongation; for the latter, the risk is mainly conditional [10]. In addition, the pharmacists’ recommendations, when present, to identify and prevent QT interval prolongation were mainly focused on drugs rather than patients’ other risk factors. 

Previous studies have shown low awareness of the risk of TdP among healthcare professionals [13,14], which also seemed to be the case here. A recent study successfully tested the effectiveness of an e-learning program for community pharmacists about the risks of QT interval prolongation, that can assist pharmacists in identifying and preventing this uncommon but dangerous adverse drug effect [15]. Studies have also successfully shown that, with appropriate decision aids, pharmacists are capable of supporting physicians in the prevention and monitoring of QT interval prolongation [16,17]. 

This study had a few limitations. Since the study was retrospective, the research team was not able to check the validity of the recorded data. In some HMR reports, data for the risk calculation was not available, especially regarding the pathology data. That might have led to an underestimation of the prevalence of risk factors (e.g., electrolyte imbalances or co-morbidities) since patients without the recorded data were categorized as not having the risk factor. The CredibleMeds^®^ drug list is updated when new evidence regarding a drug’s potential to induce QT interval prolongation is identified. The reports included in this study were written over a period of four years. This means that at the point of the medication review, a drug might not have been listed. This limitation was minimised by using a list with status as of 2015.

The findings of this research could stimulate the development of an action plan for pharmacists to support prescribers to better prevent the occurrence of drug-associated TdP and potentially sudden cardiac death. An education module about QT interval prolongation could be implemented in pharmacy undergraduate and continuing professional education programs. Other authors have also called for more intense training in cardiac safety at pharmacy schools [18].

## 5. Conclusions

There is an appreciable risk of drug-induced QT interval prolongation among patients undergoing medication reviews. Furthermore, the presence of other risk factors, such as older age and gender, puts many patients at additional risk of this adverse event. Pharmacists’ recommendations in medication reviews, when present, were targeted on drugs with known risk of QT interval prolongation, rather than patients’ additional risk factors. There is a need to improve knowledge and awareness of this topic amongst pharmacists performing medication reviews.

## Figures and Tables

**Table 1:** Examples of drugs with risk of QT interval prolongation and torsade de pointes (TdP) [10].

Category	Known Risk	Possible Risk	Conditional Risk
Definition	“… prolong the QT interval AND are clearly associated with a known risk of TdP, even when taken as recommended.”	“… can cause QT prolongation BUT currently lack evidence for a risk of TdP when taken as recommended.”	“… associated with TdP BUT only under certain circumstances of their use OR by creating conditions that facilitate or induce TdP.”
Examples	Amiodarone	Aripiprazole	Amitriptyline
	Azithromycin	Buprenorphine	Furosemide
	Chlorpromazine	Clozapine	Hydrochlorothiazide
	Ciprofloxacin	Imipramine	Indapamide
	(Es-)Citalopram	Lithium	Loperamide
	Domperidone	Mirtazapine	Pantoprazole
	Fluconazole	Ofloxacin	Quetiapine
	Sotalol	Venlafaxine	Sertraline

**Table 2:** Risk factors for QT interval prolongation and their corresponding multiplier (according to RISQ-PATH score [12]).

Risk Factor	Multiplier
Age ≥65 years old	3.0
Female gender	3.0
Smoking status	3.0
BMI ≥30 kg/m^2^	1.0
Cardiomyopathy	3.0
Hypertension	3.0
Arrhythmia	3.0
Existing prolonged QT interval	6.0
Thyroid disturbances	3.0
Liver failure	1.0
Neurological disorders (stroke, tumour, infection, trauma)	0.5
Diabetes	0.5
Hypokalaemia (≤3.5 mmol/L)	6.0
Hypocalcaemia (<2.15 mmol/L)	3.0
Inflammation (CRP >5 mg/L)	1.0
Renal impairment (eGFR ≤30 mL/min/1.73 m^2^)	0.5
Known risk QT interval-prolonging drug	3.0 per drug
Possible risk QT interval-prolonging drug	0.5 per drug
Conditional risk QT interval-prolonging drug	0.25 per drug

Abbreviations: BMI = body mass index, CRP = C-reactive protein, eGFR = estimated glomerular filtration rate.

**Table 3:** Baseline characteristics of patients taking QT interval-prolonging drugs (*n* = 325) and commonly taken QT interval-prolonging medication (*n* = 459).

Baseline Characteristics of Patients	Mean ± SD (Range) or *n* (%)	
Age (years)	76 ± 12 (20–97)	
Medications/supplements per patient	10 ± 3 (2–20)	
Chronic medical conditions per patient ^a^	6 ± 3 (1–15)	
Female	211 (64.9)	
**QT Interval-Prolonging Drug**	**Patients *n* (%)**	**Risk**
Hydrochlorothiazide	90 (19.6)	Conditional
Pantoprazole	89 (19.4)	Conditional
Furosemide	85 (18.5)	Conditional
Indapamide	36 (7.8)	Conditional
Sertraline	31 (6.8)	Conditional
Amitriptyline	23 (5.0)	Conditional
Mirtazapine	21 (4.6)	Possible
Metoclopramide	18 (3.9)	Conditional
Venlafaxine	15 (3.3)	Possible
Citalopram	13 (2.8)	Known
Escitalopram	13 (2.8)	Known

^a^ data available for *n* = 312; Abbreviation: SD = standard deviation.
